# The primary cilium regulates the expression and subcellular localization of kinesin-3 family member Kif13B

**DOI:** 10.1186/2046-2530-1-S1-P37

**Published:** 2012-11-16

**Authors:** LB Pedersen, RI Dinant, A Aleliunaite, IR Veland, ST Christensen

**Affiliations:** 1 Department of Biology, University of Copenhagen, Denmark

## 

In *Caenorhabditis elegans* the kinesin-3 Klp6 is required for localization of polycystin-2 to sensory cilia (Peden and Barr, 2005) and regulation of IFT (Morsci and Barr, 2011). A kinesin-3-encoding gene from *Drosophila* contains a consensus binding site for cilia-specific RFX-type transcription factors, suggesting a potential ciliary function for this kinesin (Laurencon et al., 2007). Furthermore, the kinesin-3 Kif13A localizes to centrosomes in HeLa cells (Sagona et al., 2010), but if any mammalian kinesin-3 family proteins play a role in ciliary assembly or function is unknown. We recently showed that kinesin-3 Kif13B is upregulated during growth arrest in NIH3T3 cells, suggesting potential cilia-related functions for Kif13B (Thorsteinsson et al., 2009). Further, we showed that the primary cilium plays a critical role in regulating directional cell migration in mouse fibroblasts (Schneider et al., 2010). Here we report that endogenous and GFP-fused Kif13B are localized to the base of the primary cilium, as well as to cell-cell adhesion sites, in growth-arrested mouse fibroblasts and other cultured mammalian cell types. This localization is abolished in *Tg737orpk* mouse embryonic fibroblasts (MEFs), which lack primary cilia, and in *inv-/-* MEFs, which are defective in cilia-mediated Wingless/Int signalling, cell polarity, and directional cell migration. Using RT-qPCR we show that the expression level of Kif13B is dramatically reduced in *Tg737orpk* MEFs compared to wild type MEFs, indicating that ciliary signalling controls the cellular level and localization of Kif13B. We hypothesize that KIF13B functions downstream of the cilium to promote cell polarity and directional cell migration.

